# Arbitrary mangrove-to-water ratios imposed on shrimp farmers in Vietnam contradict with the aims of sustainable forest management

**DOI:** 10.1186/s40064-016-2070-3

**Published:** 2016-04-12

**Authors:** Urs Baumgartner, Shelagh Kell, Tuan Hoang Nguyen

**Affiliations:** Ekolibrium, Hohrainstrasse 5, 3256 Bangerten, Switzerland; School of Biosciences, College of Life and Environmental Sciences, University of Birmingham, Edgbaston, Birmingham, B15 2TT UK; Ho Chi Minh City Open University, Ho Chi Minh City, Vietnam

**Keywords:** Shrimp–mangrove farming, Sustainable development, Environmental policy, Sustainable livelihoods, Eco certification, Natural resource management

## Abstract

Worldwide, an estimated 35 % of mangrove forests have been lost between 1980 and 2005—among other reasons due to expansion of aquaculture production systems. In Vietnam, where the total mangrove area decreased from 269,150 ha in 1980 to 157,500 ha in 2000, regulation of such systems in the form of ‘mangrove-to-water surface ratio’ has had limited success to halt these losses. In this study, a survey of 40 Vietnamese households was conducted in mangrove production forests in Rach Goc commune, Ngoc Hien district, Ca Mau province to understand whether fixed limits on minimal mangrove coverage influence farmers’ decisions on mangrove protection. Results of the survey suggest that rural households greatly depend on the incomes generated from shrimp (and crab) farming but that they do not have a share in economic incentives from timber harvests due to lack of full ownership. A strong relationship between mangrove coverage and per pond area income was also revealed. Because farmers are not aware of applicable laws in terms of mangrove-to-water ratios, mangrove coverage tends to shift in favour of higher pond areas. Overall, the findings indicate that regulations in the form of universal mangrove-to-water ratios do not consider the realities of local households, nor are they economically or environmentally useful—rather, they appear to be arbitrary limitations that are not respected by affected communities. The findings question the efficiency of efforts put into stricter enforcement.

**Electronic supplementary material:**

The online version of this article (doi:10.1186/s40064-016-2070-3) contains supplementary material, which is available to authorized users.

## Introduction

Mangrove forests provide a variety of ecosystem services, including climate change mitigation and food for local communities. Worldwide, an estimated 35 % of mangrove has been lost between 1980 and 2005 (MA 2005). In Vietnam, the total mangrove area decreased from 269,150 ha in 1980 to 157,500 ha in 2000 (FAO 2007). One of the major reasons for mangrove degradation is the fast expansion of aquaculture, a form of food production known for centuries but which has only gained particular commercial importance since the 1980s, mainly as a reaction to depleted fish stocks and an increasing demand for seafood. It is estimated that on a global scale aquaculture production is responsible for more than 50 % of the overall loss of mangrove (Valiela et al. 2001).

Mixed shrimp–mangrove systems are a traditional form of raising shrimp that are markedly different to other production forms. Based on an ecosystem approach they benefit from rather than overexploit their surroundings (Hogarth 2007). Such systems can be found to a large extent in Ca Mau province, the southern-most province of Vietnam, where so-called production forests have increasingly been transformed from pure silvi-culture to mixed production systems combining forest production with shrimp cultivation. However, excessive shrimp farming led to forest degradation and required legal restrictions on mangrove removal. In 2002, based on national Decision 178/2001/QD-TTg (GoV 2001), the local government in Ca Mau introduced Decision 24/2002/QĐ-UB which stipulated that the use of production forests for non-timber extraction be limited to a maximum of 30 % aquaculture for farms with more than 5 ha of land, while the ratio was 60:40 for farms of between 3 and 5 ha, and 50:50 for farms with less than 3 ha (PPC 2002). The higher provincial limits were a deviation from the maximum 30 % of land use for aquaculture production stipulated in the national decision but only applied to households that had been in the area before the introduction of the new law.

Despite legal restrictions on mangrove clearing, it has repeatedly been observed that degradation of mangrove habitats continues and that overall tree coverage is much lower than the minimum levels stipulated by law, which indicates that farmers in the area do not comply with current regulations. Responding to apparent weak institutional structures and a deficient regulatory body (Ha et al. 2012a), private approaches to mangrove conservation have been put forward as more promising alternatives to a top-down approach (Thuy et al. 2013). The first project using private certification started to be implemented in Ca Mau province in the year 2000 and 2 years later, the first farms became certified according to the organic standard ‘Naturland’ (Censkowsky 2014). The number of participating farms grew steadily until 2006 when the first farmers decided to leave the programme (Ha et al. 2012a). In 2009, a second project using the ‘Naturland’ organic standard was implemented. Both projects seemed to either have stopped or largely reduced in the number of participating farmers by the end of 2013 (Brunner 2014; Censkowsky 2014). While such observations question the long-term success of ‘Naturland’ organic certification projects, the Vietnamese government has the ambitious plan to expand certification to all the mixed shrimp–mangrove farming systems along the entire coast of Ca Mau province (Ha et al. 2012b).

In contrast to the reported strengths, eco-certification has earned significant criticism when applied to extensive shrimp value chains. Ha et al. (2013) found that although a suitable model for mixed shrimp–mangrove systems, organic certification might not be sustainable when the farmers producing the products do not benefit from the respective value chains. High transaction costs for the establishment of a certification scheme have been identified as a major challenge when working with smallholders and consequently, eco-certification might marginalize small-holders from participation in export-oriented value chains (Tran et al. 2013; Marschke and Wilkings 2014).

Non-inclusive standard development processes and/or different epistemologies may result in stakeholders not fully participating and finding ways to manipulate the certification process with the consequence that the certification status does not match with the reality (Konefal and Hatanaka 2011). Third-party certification may result in a verification of whether production practices are in conformity with pre-set standards and not if the value chains achieve their claims (Konefal and Hatanaka 2011). The impacts of certification may also be limited when applied to products that already have a high level of compliance, which is the case for extensive shrimp–mangrove systems (Ha et al. 2012b). The same author found that eco-standards fail to see the big picture and narrow interpretation of the standard leads to discrimination against the smallest households. A proposal brought forward by a group of farmers aiming at certification of their small-scale production systems as one ‘large ecological unit’ and thus allowing them to benefit from the ‘Naturland’ eco-standard by meeting the minimum forest coverage of 50 % required was not accepted by ‘Naturland’, even though it would make sense from an ecological standpoint (Ha et al. 2012b). The fact that mangrove protection should be discussed at landscape level is supported by observations from Koch et al. (2009) and Polidoro et al. (2010) who found that mangrove habitats do not show linear functionalities. In other words, from an ecosystem perspective it could be that a mangrove forest of a certain size with 30 % forest coverage provides the same benefits as a mangrove forest of the same size but with 50 % coverage. Vandergeest (2007) explains that while claiming sustainability as their targets, in reality various eco-standards—‘Naturland’ organic included—do not strongly focus on environmental impacts.

Bush et al. (2013) conclude that certification is mainly a strategy for buyers seeking to avoid reputation loss and public outcry over their sourcing policies and that aquaculture standards hardly consider local characteristics. While it originally emerged as an answer to weak state regulation, eco-certification can now be blamed for being inflexible and restrictive. Even if claimed to be neutral due to techno-scientific characteristics that make it independent, measureable and verifiable, third-party certification is not culturally neutral and standards based on Western norms, values, and ideas of rationality may not be successfully implemented in other parts of the world, particularly not in countries of the global South (Hatanaka 2010). Vandergeest (2007) showed that if local communities are mentioned in private standards, then it is only in terms of human and labour rights, but without including local communities in the process of formulating, enforcing and monitoring of the standard. Non-inclusive processes lead to standards that do not really suit the needs of those who should apply them. However, participation of local communities is critical in shaping the landscape they live in and conservation must be balanced with the priorities of those communities (FORRU 2008). It follows that identifying impacts with strong local character and addressing those would be a key for regulation.

Today, land use for aquaculture production in production forests in Ca Mau is restricted under a national decree to a maximum of 30 % regardless of the farm size (GoV 2006a) and modified by Decision 186/2006/QD-TTg to a maximum of 40 % (GoV 2006b), while the ‘Naturland’ organic standard demands a ratio of 50:50 (Naturland 2014). The apparent goal of such limitations to mangrove exploitation is the maintenance of important ecosystem services that coastal mangrove forests provide—thus, the aim is to prevent further degradation of the affected areas. Yet, it appears that an in-depth analysis of whether fixed ratios of mangrove-to-water surface are sustainable from an ecological, socio-economic and practical viewpoint has never been carried out.

In order to provide new evidence on what mangrove management needs to consider from a farmer’s perspective, the overall aim of this study was to determine whether fixed limits on minimal mangrove coverage such as those provided by Vietnamese law or by the ‘Naturland’ organic standard are the best means to influence mixed shrimp–mangrove farmers’ decisions on mangrove protection. Our intention was to fill a knowledge gap and to make a significant contribution towards answering the question of how and under what circumstances improvement projects using eco-standards are able to secure farmer participation, which in turn is a prerequisite for certification to work successfully in the short and long term.

## Methods

**Fig. 1:**
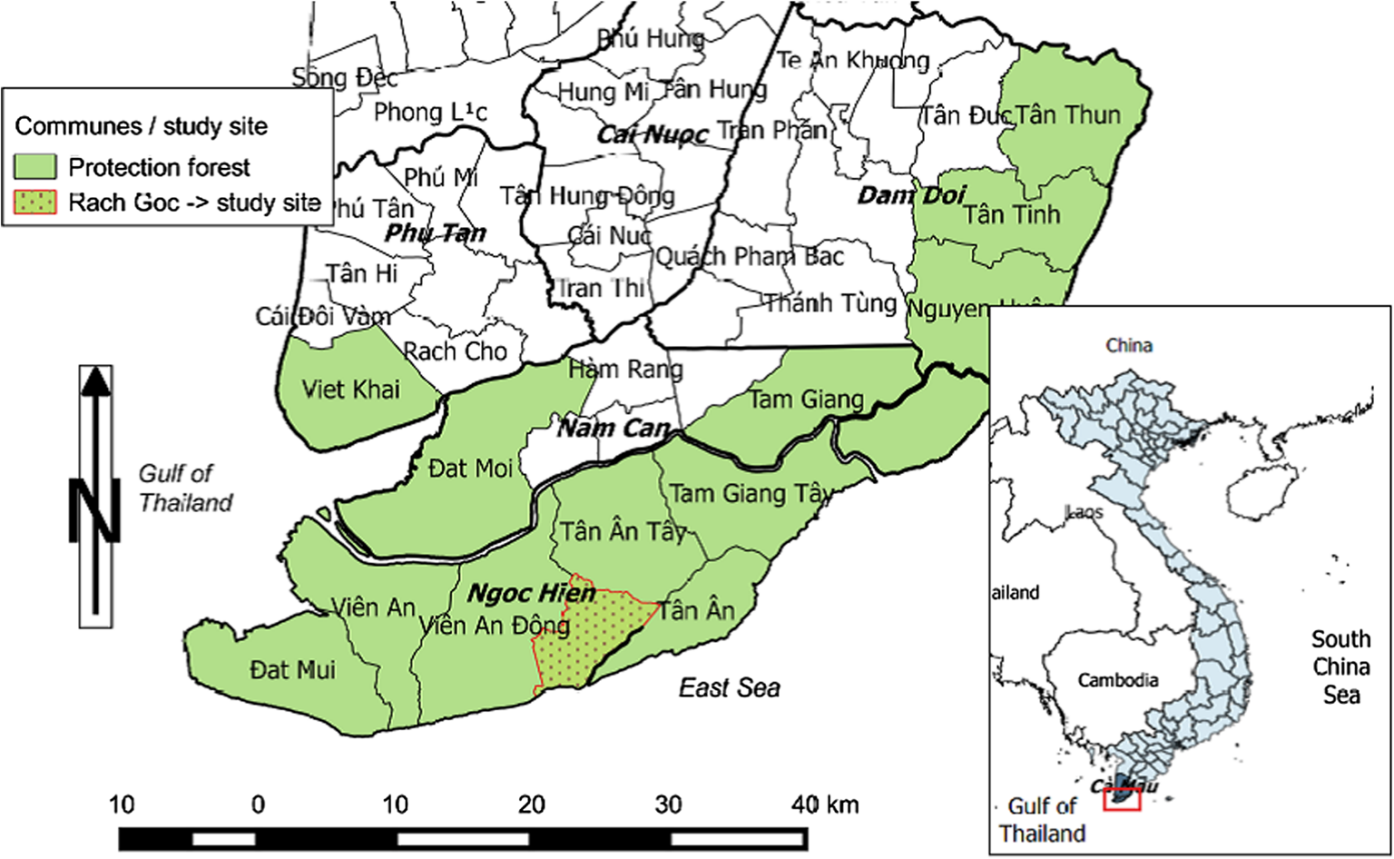
The districts and communes of Ca Mau province in which production forests occur highlighting the study site—Rach Goc commune. *Source* author adapted from data by GADM (2012)

The research involved a survey of households dedicated to shrimp farming in mixed shrimp–mangrove systems in Ca Mau province, Vietnam, where mixed shrimp farming is practiced in the production forests of fourteen communes in four different districts (Fig. 1). Field work was carried out in June 2015 in the commune Rach Goc, Ngoc Hien district.

Forty randomly chosen households practising shrimp–mangrove farming were surveyed with the objective to understand farmers’ perspectives on mandatory restrictions on forest use. Structured interviews^1^ with farmers elucidated the questions: (1) what factors influence farmers’ decisions to maintain a certain ‘mangrove-to-water surface ratio’ on their farms?; (2) what is the farmers’ preferred ‘mangrove-to-water surface ratio’?; (3) how do legal restrictions influence farmers’ decision-making on mangrove protection?; and (4) how do restrictions under an eco-standard certification scheme influence farmers’ decision-making on mangrove protection?

To gain accordant answers, we collected general household information such as number of household members, ownership type and length, income streams and amounts, and explored motives for farming, management strategies, monitoring frequencies and sanctions, actual mangrove-to-water ratios, ideal or preferred ratios, and benefits that mangrove provide. We also assessed the knowledge of households on applicable law, their perceptions on private regulation, experiences from former participation in a certification project and expectations towards potential future participation. The information gained was complemented with observations made during the field trips and with data from a number of semi-structured interviews carried out with other stakeholders between February and July 2015.

The sample frame obtained from the local Forest Management Board (FMB) contained all households (602) practising mixed shrimp–mangrove farming within a production forest in Rach Goc commune. A sample of 40 households was selected using a mix of simple random and convenience sampling. Households are accessible by boat only, therefore, landing sites were chosen using simple random sampling and one or more households in the vicinity were interviewed. The sample contained farms that had participated in a certification project using the ‘Naturland’ organic standard from 2010 until 2012 and farms that have never been certified before.

A Pearson’s product-moment correlation coefficient was computed to assess the relationship between the actual mangrove coverage and total household incomes, number of household members, per capita income, total farm size, length of ownership of the interviewed farm households, and total income per pond area. For assessing the correlation between mangrove coverage and total income per pond area, a new variable, ‘household income per pond area’ was created and then the results transformed (ln) before testing for correlation. One-way between groups analysis of variance (ANOVA) tests were used to check if former participants in an organic project and non-participants show differences in terms of actual mangrove coverage (self-reported and reported by FMB), perceived ideal mangrove coverage, perceived productivity of their operations, compliance with legislation, or awareness thereof. In addition, qualitative information from the questionnaires and other sources of information were evaluated using ‘thematic analysis’. Data from transcripts were categorized according to emerging themes such as productivity, reforestation efforts, and climate change and themes that served to answer the research questions, amongst others compliance, benefits of mangrove, and costs and benefits of organic certification.

Maps were elaborated using Geographic Information System software QGIS and data from GADM (2012).

## Results

### General characteristics of the sample

All interviewed farmers moved to the area from elsewhere in Ca Mau or other provinces with the goal to engage in shrimp–mangrove farming. The main motivations for respondents moving to their current location were shrimp farming (80 %), expected income (57.5 %), and family connections (42.5 %). Farmers have owned or leased their farms for 1 to 45 years, with an average of 22 years and the majority (62.5 %) have lived on their farms since before 2001. The majority (85 %) of interviewed households have a ‘green book’^2^ and lease the farm from the FMB while the rest manage the farm for third parties—usually close relatives. For most households, current contracts with the FMB will expire in 2034. Fifteen (37.5 %) of the households had participated in a ‘Naturland’ organic certification project that ran from approximately 2010 until 2012 when it eventually came to an end without farmers having been informed about the exact reasons for and time of ending.

### Factors affecting farmers’ decisions on mangrove management

**Fig. 2:**
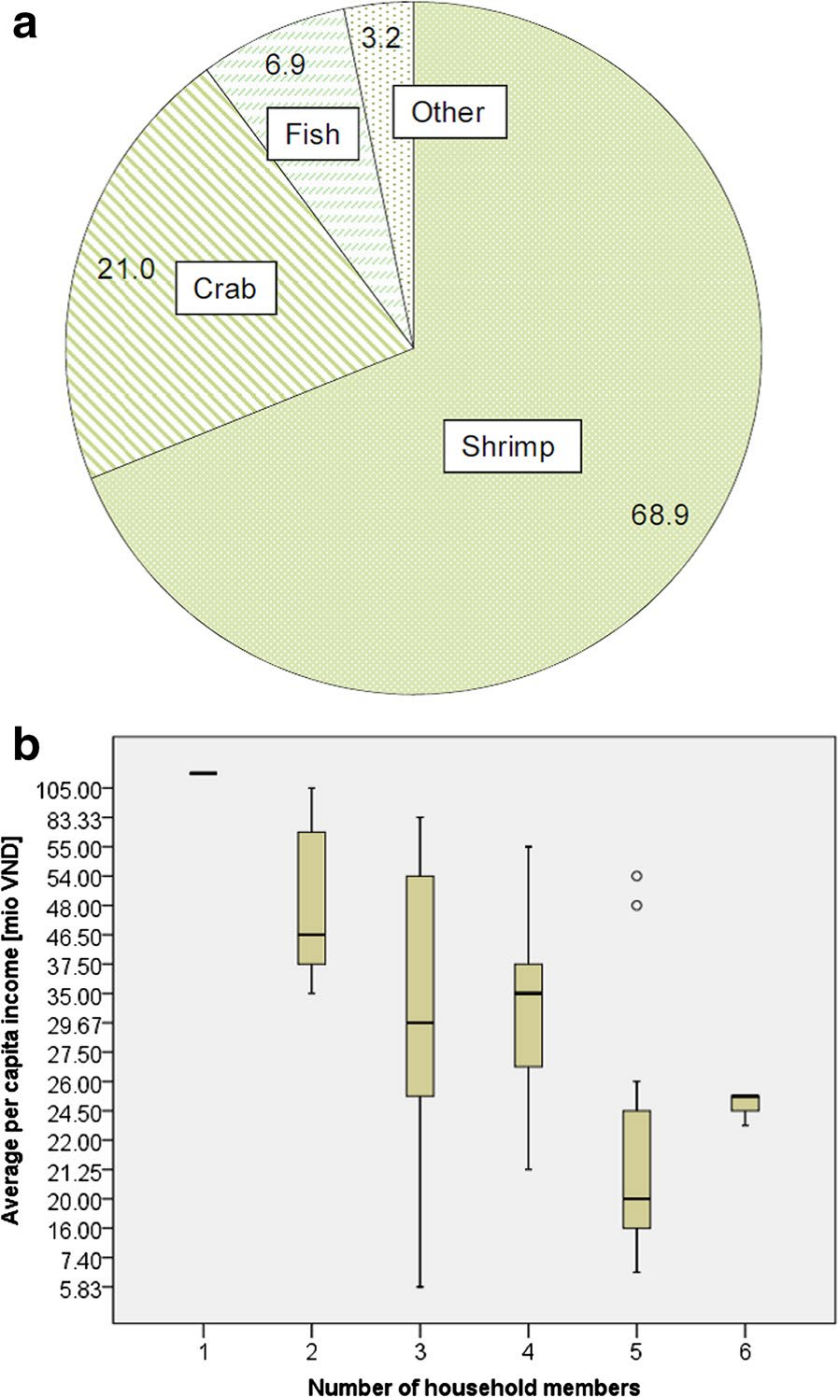
**a** Household incomes (%) according to source of income and **b** average per capita income according to number of household members (mio VND)

The main influence on farmers’ decisions regarding mangrove management is the income generated from their farming operations. Income is followed by uncomplex management and market access, while flexibility with the management system^3^ appears to be less relevant (Table 1). On average, the income from shrimp production accounts for 68.9 % of the overall household income. Together with the income from crab farming, aquaculture production makes up nearly 90 % of overall household incomes (Fig. 2a). The self-reported average income per year is 110 million VND (USD 5046^4^) for single occupancy households, gradually decreasing according to the number of household members to between 7.4 million VND (USD 340) and 26 million VND (USD 1193) for households with five or six members (Fig. 2b). Across all households, average income is 37.6 million VND (USD 1725; median = 29.0 million VND/USD 1330).

**Table 1:** Factors affecting households’ decisions on mangrove management (*n* = 40)

Factor considered	Very important (%)	Important (%)	Slightly important (%)	Not important (%)
Income from farming operations	92.5	7.5	0	0
Uncomplicated management of operations	82.5	15.0	2.5	0
Market access for harvested products	72.5	15.0	7.5	5.0
Flexibility with management system^a^	45.0	20.0	15.0	20.0

^a^E.g. to have flexible rules to mangrove-to-water ratio

### Preferred ‘mangrove-to-water surface ratio’

Most farmers (82.5 %) believe that mangrove trees are good for their farm. The benefits mentioned are nursing services (90 %), climate regulation (80 %), timber (65 %) and biodiversity (65 %). Wave protection was mentioned by 40 % of respondents and aesthetic values by 35 %. Farmers believe that mangrove trees influence the productivity of their shrimp operations, but do not exactly understand how. Some believe that the shading they provide is beneficial for shrimp. Others believe that certain tree species are good for shrimp productivity while other species have a negative impact.

**Fig. 3:**
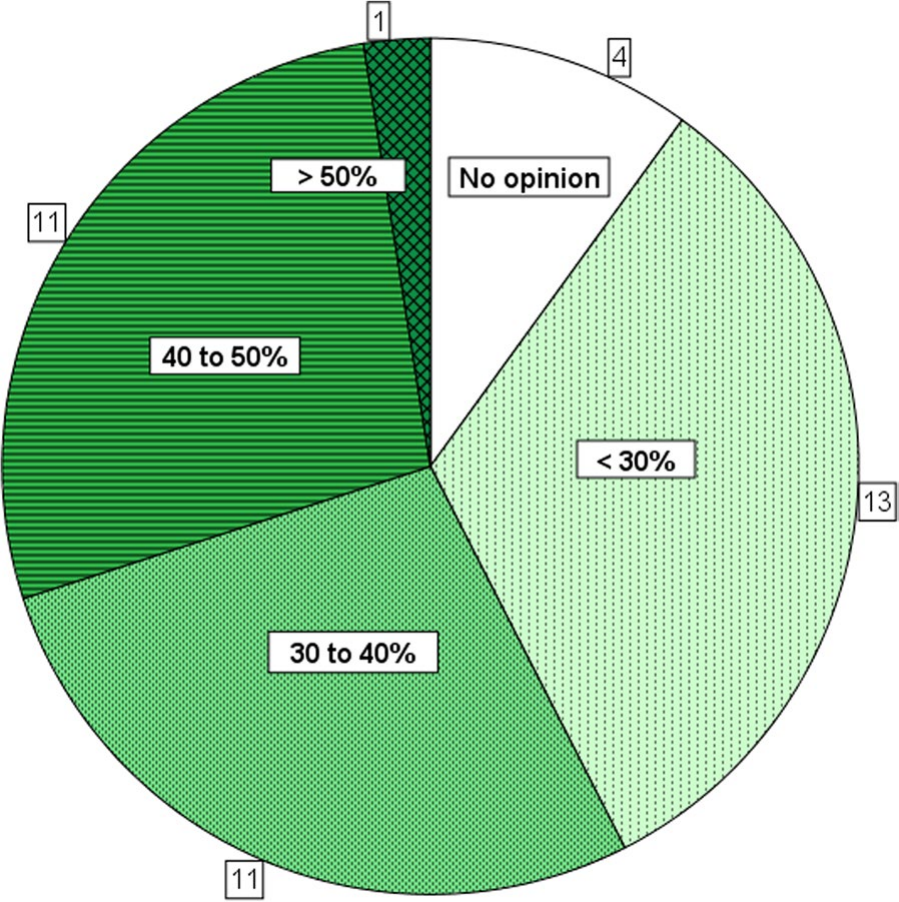
Perceived mangrove coverage that is seen as ideal by farmers (*n* = 40)

The majority of interviewed farmers believe that the best mangrove-to-water surface ratio lies somewhere between 30 and 50 % (Fig. 3). A third of respondents prefer mangrove coverage of <30 % while one out of ten have no opinion or do not know. Only one of the interviewed farmers believes that more than 50 % mangrove coverage would be good for their farm. When asked for other means to improve productivity, better post-larvae (PL),^5^ a bigger pond area, multi-species farming, and better management are mentioned most frequently (Table 2). The majority of farmers believe that higher or lower tree coverage compared to their actual ratio would not improve productivity on their farms. The ‘income per hectare pond area’ varies significantly between farms, from a low 15 million VND/ha/year to 219 million VND/ha/year.

**Table 2:** Farmers’ perceptions on productivity of their aquaculture operations (*n* = 40)

Perception on productivity	Respondents agree (%)
Bigger pond area improves productivity	80.0
Removing trees increases productivity	32.5
Planting trees increases productivity	17.5
Better PL improve productivity	85.0
Better management improves productivity	50.0

### Legal restrictions on forest use

Legal restrictions do not seem to have a big influence on farmers’ decisions regarding mangrove management. Only 15 of the interviewed farmers knew the exact legal restrictions that apply to their farming operations. All others either did not know (21) or believed to know but were wrong (4). Many respondents stated that they did not have the means to increase the pond area and therefore that coverage had always been the same and did not change over time. A majority (35) believe that they are compliant with legal norms and many feel that as long as authorities did not complain, they assume to be in conformity with applicable rules. In reality, only six of the interviewed households had a mangrove-to-water ratio of the stipulated 60:40 or above. Eight respondents have higher mangrove coverage than they believed they did, while the rest either did not know or they overestimated it.

Farms are monitored once every 3–6 months, although opinions vary or the monitoring frequency is not the same for all households. There are no real sanctions for elevated or incompliant deforestation. If such deforestation is detected, the FMB asks affected households to reforest to a mandated amount and generally supplies the seedlings for reforestation.

No correlations between actual mangrove coverage and total household incomes, number of household members, per capita income, total farm size or length of ownership were detected. In contrast, there was a strong correlation between the actual mangrove coverage of the interviewed farms and total incomes per pond area [Table 3—Pearson’s correlation: (1, N = 40) = .387, p = 0.007].

**Table 3:** Pearson correlation between mangrove coverage on farms (cover_FMB) and the total annual income per pond area (lnperpondareincome)

	cover_FMB	lnperpondareincome
cover_FMB		
Pearson correlation	1	.387^a^
Sig. (1-tailed)		.007
N	40	40
lnperpondareincome		
Pearson correlation	.387^a^	1
Sig. (1-tailed)	.007	
N	40	40

^a^Correlation is significant at the .01 level (1-tailed)

### Restrictions under an eco-standard certification scheme

Households look upon certification positively. The majority indicated that they would participate in a new certification project, while one out of three do not know or cannot decide without having further information about the project or certification scheme. Openness to join a future project does not vary between households who have participated in a former organic project and those who have not, even though 50 % of previous participants were only moderately satisfied with the former project while 42.9 % said that they were not satisfied at all. Reasons for these ratings were mixed and many farmers felt ambiguous about their participation. While ‘participation’ as such and ‘the possibility to learn’ were appreciated most, respondents felt that they did not really benefit from the former certification project. Many complained about an absence of economic incentives, lack of transparency, and unfair treatment by traders and collecting stations to whom they felt exposed. Respondents explained that the monitoring frequency did not increase or decrease under the certification scheme.

Benefits that would make future participation attractive are: access to better PL (85 %), technical support (77.5 %), formation of a farmer group (75 %), and overall higher household incomes (65 %).

Former participants in an organic project do not show significant differences to non-participants in terms of actual mangrove coverage,^6^ perceived ideal mangrove coverage, perceived productivity of their operations, compliance with legislation, or awareness thereof.

## Discussion

The results suggest that mangrove degradation in production forests is primarily a consequence of socio-economic realities. With a reported^7^ annual average of USD 1725 per capita, incomes in surveyed households are low.^8^ They are significantly lower for households with five or more members. Due to their isolation, households have no other means to generate income than from the allocated forest lots which are relatively small. Incomes from shrimp farming combined with incomes from crab farming account for almost 90 % of average household incomes. Farmers report that they do not receive any economic benefit from timber, a fact that is probably related to the lack of ownership discussed by Ha et al. (2012a). Due to the lack of ‘de facto’ ownership over timber products, farmers see no alternative than to expand their shrimp production if they want to increase household incomes. The observed expansion of shrimp production is thus a consequence of economic needs and desires. Aquaculture is a proximate cause and not the ultimate driver of deforestation, a fact that confirms established knowledge (Geist and Lambin 2005).

From an enforcement perspective, fixed limits on mangrove use cannot solve the problem of weak governance if enforcement is not funded accordingly. The findings of this research suggest that farmers do not suffer any harm if they do not comply with mandated ratios. De Jong et al. (2006) found that FMBs themselves are struggling with underfunding and hence the observed superficiality of monitoring and enforcement could directly be related to the lack of financial support. Even eco-certification cannot solve this problem due to the ease for stakeholders to deceive the certification process, as Konefal and Hatanaka (2011) have shown. This could explain why interviewed farmers who have previously participated in an organic project would participate again, even if limitations in organic standards contradict with their needs and personal preferences and even though most of them were not really satisfied with the outcomes of their previous participation. Households have nothing to lose from participation while they can always hope to gain something, no matter how small the benefit. In the case of shrimp–mangrove farms, geographic isolation, weak enforcement capacities and overall high corruption in Vietnam (Martini 2012; Dien 2014) might all contribute to a situation in which economic incentives for certified products can co-exist with further degradation of forests.^9^ It could further be reinforced by a design problem of certification schemes that generate economic incentives for shrimp products from shrimp–mangrove farming systems: higher shrimp product prices increase the attractiveness of production, and this might lead to an expansion of shrimp operations. If this happens within geographically limited areas (e.g. the same farm or the same commune), ‘cheating’ on the certification process and/or the mangrove-to-water ratio must be expected. Consequently, certification for shrimp in shrimp–mangrove systems has the potential to create perverse outcomes.

A further problem of enforcement of mangrove-to-water ratios appears to be communication. Legal restrictions have changed over time and the fact that most households do not even know the legal basis mandating the management of their operations suggests that incompliance is an almost imperative consequence. Due to the lack of ownership and understanding, quantitative restrictions on mangrove forests are however not respected by farmers. While farmers recognize that mangrove trees benefit their farming operations in different ways, they believe that mangrove-to-water ratios of between 30 and 50 % bring more benefits for them than higher tree coverage, an observation that is in line with those made by Binh et al. (1997) who found that ponds with mangrove coverage of 30–50 % yield the highest incomes for farmers, while shrimp yields decrease as mangroves grow, suggesting that mangrove density has a negative impact on shrimp yields. The stipulated mangrove-to-water ratios of 60:40 seem thus to contradict with productivity.

In this regard, the finding that actual mangrove coverage of interviewed households is related to the annual income per hectare of pond surface is of upmost importance, although the results should be considered with caution due to the small sample size and the fact that self-reported incomes might not fully reflect the realities. The incomes per hectare of pond area are an indicator of productivity and they vary significantly between households, which might confirm observations made by Ha et al. (2013) that productivity is very different among farms. If higher productivity leads to better incomes and reduces the pressure on households to further expand their pond areas, improvements should focus on this aspect. Earlier works and feedbacks from farmers suggest that farm management, different tree combinations, better PL, and better pest control could potentially increase productivity of present farming systems. Optimized productivity by maintaining full ecosystem services could thus be used as an angle for conservation and there is room for further investigation into this topic.

This observation challenges the usefulness of fixed mangrove-to-water ratios, which must also be doubted from an ecosystem perspective. Fixed limits as under the ‘Naturland’ organic shrimp standard or Vietnamese law only superficially address ecological aspects. Ecosystems do not show linear characteristics except for above-ground carbon sequestration (Koch et al. 2009) and simple mangrove-to-water ratios are thus not an indicator for habitat quality or biodiversity.

In fact, it appears that such ratios are a historic relict rather than a rational threshold. Although not fully confirmed, it appears that the ratio for mangrove-to-water surface that was introduced as a law in the years 2000/2001 is closely related to the first organic shrimp project in the province which was implemented by Institute for Marketecology (IMO), Swiss Import Promotion Programme (SIPPO) and Naturland in 2002, and that used a custom-made standard based on the very first ‘Naturland’ organic standard for shrimp which had been published in 1999 (IMO 2002). It mandated a ratio of 50:50 for pond-based production and 70:30 for ‘integrated’ shrimp production in mangrove. According to Naturland [Wiedenlübbert U., personal communication, Boston (MA), 17 March 2015] the minimal ratio for mangrove coverage in the first ‘Naturland’ standard for shrimp had been the outcome of a stakeholder meeting in 1997 in Ecuador between members of shrimp producers, representatives from ‘Naturland’ as the standard setting body, auditors from certification bodies, and other experts. The limitations on mangrove clearing were based on a precautionary approach, the situation and knowledge about mangrove at that time, addressing post-Rio forest agendas, and considering shrimp farming systems in Ecuador. Given the close relation between the mangrove-to-water ratios in both regulations it can thus be argued that limitations in the ‘Naturland’ organic standard as much as in Vietnamese law root in a non-science-based but rather situative decision taken in the late 1990s considering the characteristics of large-scale shrimp farms in Ecuador, which are very different from the integrated small-holder systems in Ca Mau. Universal in their approach, mangrove-to-water ratios do not consider the realities of affected communities in Vietnam, which live in and from mangrove forests. Their relationship to and management of mangrove is in stark contrast to large companies buying mangrove forest in Ecuador in order to transform them to large-scale shrimp farms.

Eventually, restrictions on forest exploitation must also be discussed from a justice perspective. Coastal forests in Ca Mau have been appreciated for their ecosystem services at least since the time when Vietnam was under French colonial powers (Hong and San 1993) and forest management has ever since been based on economic decisions. The first settlers moved there in the 1950s due to threat of famine in the Mekong Delta further north. Until today, local communities in production forests have remained relatively poor. The majority of interviewed farmers came to live in Ca Mau with the main objective to farm shrimp, most of them at a time when use restrictions on mangrove were different from today or did not exist at all. The prevailing conditions at that time created expectations. Over time, legislation and enforcement changed, not least due to changing global political agendas and international initiatives such as REDD, which put forests—and more specifically mangrove forests—on international radars, often with a narrow focus on only one of many ecosystem services that mangrove provide, the capacity of forests to sequester carbon dioxide from the atmosphere and thus to reduce human induced climate change. Such narrow views fail to see the full picture, not only in terms of climate change policies but also with regard to the interplay between ecological and social dimensions of forests. Different authors have warned that use restrictions on forests have the inherent risk of discriminating against communities living in and from forests, while local elites capture the benefits. Ha et al. (2012b) indicated that the same happened with earlier organic shrimp certification programs for shrimp–mangrove systems in Ca Mau and the unfair and untransparent payment terms in a former certification project mentioned by some interviewees might confirm that. It must therefore be questioned if certification schemes such as ‘Naturland’ organic can be justified as a way of livelihood improvement in production forests in Ca Mau. Rather, it could be that such standards have been used because they are simple to implement and monitor. Shrimp–mangrove systems in production forests make an easy target for improvement projects using certification as a tool for benchmarking, regardless of whether they discriminate against local communities or improve their livelihoods.

In contrast to other coastal areas, mangrove forests in Ca Mau have survived until today because of existing frameworks and institutions. Key actors in this process have been local communities and the mixed shrimp–mangrove farming systems that create livelihoods while also maintaining mangrove forests and the ecosystem services they provide. Mixed shrimp–mangrove farming systems have successfully combined the apparent contradiction of aquaculture production and landscape conservation. In some way they are a success story of mangrove conservation and have sustained the pressure of aquaculture expansion even if certain degradation in some areas can be observed.

It must thus be questioned if capacities are best used if invested for stricter enforcement of questionable mangrove-to-forest ratios. Improvement projects might be more successful if they tried to incorporate the needs of local people—for example by optimizing productivity while maintaining ecosystem functionality of affected shrimp–mangrove farming systems—or by protecting other mangrove areas that have been further degraded than those in Ca Mau’s production forests or which have vanished altogether. Such areas could profit from a knowledge transfer and the implementation of shrimp–mangrove farming systems, which could address the socio-economic needs of coastal communities while at the same time restoring degraded ecosystems. For the environment and seen from a broader perspective this would most likely be more beneficial than a questionable ‘improvement’ of already protected mangrove ecosystems.

## Conclusion

The present study set out to investigate what factors influence farmers’ decisions on mangrove management, what ‘mangrove-to-water surface ratio’ farmers prefer, and how legal or voluntary restrictions influence farmers’ decision-making on mangrove protection. Although the chosen sample is rather small and limited to households in one out of fourteen communes, we believe that the results are representative for the wider area due to identical farming systems and similar sizes of the respective farms of each individual household. Therefore, while generalizations can be justified, they should be considered with caution.

The findings suggest that farmers in production forests base their decisions on mangrove management mainly on economic outcomes. Due to lack of ownership on timber harvests, the main incomes of local households are generated from shrimp and crab farming. Therefore and in spite of legal restrictions, mangrove-to-water ratios tend to shift in favour of higher water surfaces, which has led to the observed forest degradation.

Legal restrictions do not change farmers’ attitudes towards mangrove protection because meeting them would be harmful for farmers’ livelihoods, while incompliance does not bear serious risks. In other words, the benefits of disregarding legal restrictions outweigh the potential costs. Farmers believe that mangrove-to-water ratios of between 30 and 50 % bring more benefits for them than the stipulated ratios of 60:40 under Vietnamese law or the 50:50 under the ‘Naturland’ organic shrimp standard. Based on a decision taken in Ecuador in the late 1990s and addressing a completely different setting, fixed mangrove-to-water ratios do not consider the needs of local communities in Ca Mau, who expand shrimp production as a consequence of lack of choices to generate alternative incomes.

It appears that fixed mangrove-to-water ratios are arbitrary and that private approaches to forest management in Ca Mau have failed because farmers did not become partners of certification. Enforcement of stricter mangrove-to-water ratios in the studied production forests appears to discriminate against forest communities and standard application exposes the same deficiencies that Purbawiyatna and Simula (2008) found in forest certification, namely that universal standards fail to consider the functions of forests in society.

Ultimately, while ‘Naturland’ organic standards might help implementing parties to harvest the “low hanging fruits of certification” that Marschke and Wilkings (2014, p. 205) described, they are not refined enough to tackle the complex interplay of conservation of ecosystem services, livelihood improvement, institutional capacity building, and the development of more equitable and participatory approaches necessary for successful natural resource management as aimed for under the umbrella of sustainable forest management. In light of the ambitious plans of the Vietnamese government to expand organic certification to all the mixed shrimp–mangrove farming systems along the entire coast of Ca Mau province, these findings are important. Projects trying to protect mangrove forests by simplistic and poorly implemented mangrove-to-water surface ratios cannot be justified on social, economic, or on environmental grounds, and if efforts to avoid mangrove degradation and deforestation in Ca Mau truly strive for a change, then the underlying causes must be addressed with a broader view and more inclusive approaches than apparently has been done so far.

## Additional files

10.1186/s40064-016-2070-3 In addition to the methodology described, the English version of the questionnaires used for the interviews with rural households in Ca Mau is presented.
10.1186/s40064-016-2070-3 In addition to the methodology described, the Vietnamese version of the questionnaires used for the interviews with rural households in Ca Mau is presented.

## Footnotes

1 For questionnaires used for the interviews refer to Additional File 1 (English version) and Additional File 2 (Vietnamese version).

2 There are two types of ownership: ‘green book’ is a lease for 20 years from the FMB with accordant restrictions on timber management and benefit sharing while ‘red book’ is a 50 years lease with full legal ownership (Ha et al. 2012a).

3 E.g. to have flexible rules to mangrove coverage.

4 Exchange rate USD: VND = 1: 21,800 on 15 June 2015.

5 A stage in the life-cycle of shrimp, generally used for stocking in shrimp production.

6 Self-reported and reported by FMB.

7 The figures are self-reported.

8 Although similar to the USD 1740 Vietnamese average per capita income estimated by the World Bank for 2013 (http://data.worldbank.org/country/vietnam).

9 This assumption is strengthened by claims of the majority of interviewed farmers who state to be compliant with applicable law, while their forest coverage was well below the 60 % and even below the 50 % threshold.
