# Supplementary material for: Arbitrary mangrove-to-water ratios imposed on shrimp farmers in Vietnam contradict with the aims of sustainable forest management
Source: Springerplus. 2016 Apr 12;5:438. doi: 10.1186/s40064-016-2070-3 (PMC4828363; doi:10.1186/s40064-016-2070-3)
Supplement: Supplementary file 1 — 10.1186/s40064-016-2070-3 In addition to the methodology described, the English version of the questionnaires used for the interviews with rural households in Ca Mau is presented. [file 40064_2016_2070_MOESM1_ESM.pdf]

Questionnaire for shrimp-mangrove farming survey

Informed consent statement

(-> Read the following text, point by point, and explain in detail if requested or unclear)

This research is undertaken in the context of a Master of Science dissertation for the University of London. Its aim is to understand shrimp-mangrove farmers' opinions on shrimp farming and restrictions on the use and exploitation of mangrove as stipulated by Vietnamese law or voluntary regulations.

1. Participation in the survey is **voluntary**.
2. **Refusal** to participate **does not have any negative consequences** (e.g. you can participate in any other survey or project without having to participate in this interview)!
3. If you agree to participate, you have the **right to ask questions or refuse further participation** at any time during or after the interview.
4. The information you share will be used and might be published. However, **your name will not appear in any form and nobody will know that you participated or what your answers were**.
5. Your information and answers are exclusively for this study and **will not be shared** with any other organization/person.

(-> Once you have read the information, make a pause. Then ask if interview participants have questions. If not, proceed.)

If you wish to participate, then please confirm this with a clear **"Yes, I do want to participate in this survey."**

(-> Wait for answer/confirmation)

Thank you very much. We would now like to ask you a few questions. Please interrupt and clarify when something is unclear. Try to answer as accurately as possible.

Farm ownership and characteristics

1. Farming system (integrated/separated)

☐ integrated ☐ separated ☐ other___________________________________

2. Farm size

___________________ ha

3. Since when have you managed/owned this farm?

Month, year _____________________(e.g. March 2002)

4. Ownership

☐ green book ☐ red book ☐ certificate of ownership but not yet official

☐ managing for third-party ☐ other____

5. When does your actual contract expire? *(e.g. until when is the green/red book issued)*

Month, year ____________________(e.g. March 2017)

6. When you started to manage this farm, what was (were) the main reason(s)? *(multiple answers possible)*

☐ shrimp farming ☐ forest management ☐ family reunification/relatives ☐ source of income

☐ other____

7. What changes to the farming system have you made since you started to manage this farm (e.g., surface area, forest coverage)? *(multiple answers possible)*

☐ no changes ☐ reforestation ☐ deforestation ☐ change between separated/integrated system
☐ increased pond area ☐ other____

8. What were the reasons for the changes undertaken? *(multiple answers possible)*

☐ increased shrimp pond surface ☐easier management ☐ optimized production

☐ regulations changed ☐ higher tree coverage is beneficial ☐conservation of mangrove

☐ other____

Mangrove management

9. What is the approximate actual tree coverage on your farm?

_______________% or _______________ha of total of ___________________ha

10. What is the legal norm in terms of minimal mangrove tree coverage on your farm?

☐ I don't know ☐ I know, it is__________________% of total area

11. Do you comply with this norm?

☐ yes ☐ no

12. How often do you get monitored (controlled) by authorities?

one time or more every: ☐ month ☐ three months ☐ six months ☐ year

☐ less than one time/year

13. What happens if authorities detect that mangrove coverage is too low? *(multiple answers ok)*
☐ nothing ☐ need to pay a fine ☐ need to reforest ☐ authorities reforest

☐ other____

(-> if they need to pay a fine, try to find out how high it is or how compensation works. Be sensitive and cautious. Amount: ____VND)

14. ***What percentage of mangrove tree coverage would be best for you if you had free choice?***

_______________% or _______________ha of total of ___________________ha

15. ***Why do you think this is the best?*** *(multiple answers possible)*

☐ highest overall income ☐shrimp production is highest ☐ provides the income I need for living

☐ optimized productivity ☐ easiest management ☐ good balance between shading to pond area

☐ best for environment ☐ good balance between different aspects (e.g. income and environment)

16. ***Would you change the mangrove coverage if the ownership changed (e.g. from green to red book)?***

☐ no ☐ yes -> *please explain: by ticking one or more of the options below*

☐ long-term conservation of mangrove ☐ I can make more profit from wood production

☐ stronger negotiation power ☐ other____

17. ***Do you think that mangrove trees are good for your farm?***

☐ yes ☐ no

please explain why____

18. ***What (other) benefits do mangrove trees provide?***

☐ protection from waves ☐ biodiversity (shelter for animals and plants) ☐ nothing/useless

☐ climate regulation ☐ nursing ground for fish/shrimp ☐ timber and other goods

☐ beauty/identity/home ☐ other ____

19. ***By law you need to maintain at least 50% mangrove. What do you think about this limit of 50%?*** *(multiple answers possible)*

☐ it is good ☐ only good for the environment ☐ it is arbitrary/random ☐ it is <u>not</u> good

☐ not important, just a law ☐ it helps officials to discriminate against farmers

Productivity

20. ***What is the income from products harvested?*** *(-> ask per year or cycle; calculate with farmer)*

☐ Black Tiger shrimp ____________VND _______________ / __________________VND per year

☐ Crab ____________VND _______________ / __________________VND per year

☐ Fish ____________VND _______________ / __________________VND per year

☐ Timber/wood ____________VND _______________ / __________________VND per year

☐ other ____________VND _______________ / __________________VND per year

21. How would you rate the productivity of your shrimp farming system?

☐ optimal (can't improve) ☐ very high ☐ high

☐ average ☐ low ☐ very low

22. Would you like to improve the shrimp productivity of your farm?

☐ yes ☐ no

23. How could you improve the shrimp productivity of your farm?

☐ bigger pond size ☐ cut more trees ☐ plant more trees ☐ better post-larvae (PL) quality

☐ other species (e.g. crab) ☐ improve management (e.g. different stocking cycles)

☐ other____

Participation in Naturland organic or similar project and outlook into future

24. Did you participate in a Naturland organic project (e.g. Seanamico) before?

☐ yes ☐ no *-> go to question 28*

25. ***During what period did you participate in the project?***

Started in ____(month, year)

certified in ____(month, year)

left project in ____(month, year)

26. How satisfied with the project were you?

☐ very much ☐ more than average ☐ average ☐ not much ☐ not at all

27. a) What are the reasons for this rating? *(-> in question 26; multiple answers possible)*

☐ economic benefits were good ☐ costs were bigger than benefits ☐ no additional benefits

☐ adaption was easy ☐ management too difficult ☐ too much paper work for certification

☐ I could learn something new ☐ I like to participate in a project/group

☐ other____

b) Controls (with project) were…… ……frequent *(-> tick one)*

☐ more ☐ about the same ☐ less

28. Would you join another / a Naturland organic or similar project?

☐ yes ☐ no ☐ maybe/not sure ☐ I need to know more to decide

29. What would make the participation in such a project attractive for you? *(multiple answers possible)*

☐ higher shrimp prices, even if less pond area ☐ no change in mangrove coverage

☐ technical support in farming ☐ access to better PLs ☐ formation of a farmer group

☐ overall higher household income ☐ participation enables access to microcredit (pay-back)

☐ other____

Outlook into the future

30. How important are the following factors to you?

Income	☐ very important	☐ important	☐ slightly important	☐ not important
(Easy) management	☐ very important	☐ important	☐ slightly important	☐ not important
(Safe) market access	☐ very important	☐ important	☐ slightly important	☐ not important
Flexibility (e.g. mangrove coverage)	☐ very important	☐ important	☐ slightly important	☐ not important

31. Where do you see yourself in 20 years' time? *(multiple answers possible)*

☐ still here ☐ I don't know ☐ not here anymore

☐ other/explain____

32. Would you want (one of) your children to take over your farm in the future?

☐ yes ☐ no ☐ I prefer them to study and work elsewhere ☐ depends on their decision

33. Do you know what climate change refers to?

☐ yes, I know very well ☐ no ☐ maybe/not sure/have some slight idea

34. Have you ever been informed about climate change and its effects by authorities or the local government?

☐ yes (please specify____) ☐ no

35. Do you believe that climate change will (or does already) affect your farming operations in the future?

☐ yes, it will have negative impact(s) ☐ yes, it will have positive impact(s)

☐ no, it will not affect my farm ☐ not sure / maybe /not important

36. Do you believe that the Vietnamese government and local authorities take appropriate measures to mitigate the negative effects of climate change to the best possible extent?

☐ yes ☐ no ☐ not sure / maybe / I don't know

Personal information (optional)

-> Please note and inform participants that this information is not necessary for the study. It might provide additional information and help to identify farmers in case of doubts/questions. However, if participants prefer not to tell, then stop here.

Do you have any questions? *(-> ask if they have any question or need clarification)*
Can we ask some personal information for statistical reasons and for clarifications if we have any questions later on? This is absolutely voluntary and you do not need to provide this information.

Thank you very much for your participation!

37. ***Name***:____

38. ***Age***: ______________________________________years

39. ***Gender***: __________________________

40. ***Total number of household members*** *(head included)*:___________________________

41. ***Phone Nr.:*** ____

42. ***Parcel Nr***.: ____

Internal

Name of interviewer_______________________________________ **Date**: ___________________________

Forest coverage as per information **FMB**:________________% or __________ha of total_____________ha

Additional comments/remarks:
