# Supplementary material for: Arbitrary mangrove-to-water ratios imposed on shrimp farmers in Vietnam contradict with the aims of sustainable forest management
Source: Springerplus. 2016 Apr 12;5:438. doi: 10.1186/s40064-016-2070-3 (PMC4828363; doi:10.1186/s40064-016-2070-3)
Supplement: Supplementary file 2 — 10.1186/s40064-016-2070-3 In addition to the methodology described, the Vietnamese version of the questionnaires used for the interviews with rural households in Ca Mau is presented. [file 40064_2016_2070_MOESM2_ESM.pdf]

Câu hỏi khảo sát nông hộ nuôi tôm rừng ngập mặn

Biên bản chấp thuận

(-> Đọc đoạn văn bản sau, từng điểm một, và giải thích kỹ nếu có yêu cầu hay chưa rõ)

Khảo sát này được thực hiện trong khuôn khổ dự án Selva shrimp thực hiện bởi Tập đoàn Thuỷ sản Minh Phú và Công ty tư vấn Blueyou. Mục đích của nó là để tìm hiểu quan điểm của nông dân nuôi tôm-rừng ngập mặn về việc nuôi tôm và các hạn chế về việc sử dụng và khai thác rừng ngập mặn theo quy định của pháp luật Việt Nam hoặc các quy chế tự nguyện.

1. Sự tham gia khảo sát là **tự nguyện**.
2. **Từ chối** tham gia **không gặp bất kỳ hệ quả tiêu cực nào** (ví dụ như ông/bà có thể tham gia vào bất kỳ cuộc khảo sát hay dự án khác mà không cần phải tham gia vào khảo sát này)
3. Nếu ông/bà đồng ý tham gia, ông/bà có **quyền đặt câu hỏi hoặc từ chối tiếp tục tham gia** vào bất cứ lúc nào trong và sau khi phỏng vấn.
4. Thông tin ông/bà chia sẻ sẽ được sử dụng và có thể được công bố. Tuy nhiên, **tên của ông/bà sẽ không** xuất hiện trong bất kỳ văn bản nào và **không ai biết rằng** ông/bà đã tham gia hay các câu trả lời của ông/bà là gì.
5. Các thông tin và các trả lời của ông/bà chỉ dành cho khảo sát này và **sẽ không được chia sẽ** với bất kỳ tổ chức/cá nhân nào khác.

(-> Khi đọc thông tin hãy dừng ở từng ý. Sau đó hỏi người được phỏng vấn xem họ có câu hỏi nào không. Nếu không có thì tiếp tục.)

Nếu ông/bà đồng ý tham gia, vui lòng xác nhận bằng câu **"Tôi đồng ý tham gia vào khảo sát này"**.

(-> Đợi cho trả lời/xác nhận.)

Cám ơn ông/bà rất nhiều. Chúng tôi muốn hỏi ông/bà một vài câu hỏi. Vui lòng hỏi ngay khi có điều gì chưa rõ. Cố gắng trả lời càng chính xác càng tốt.

Quyền sở hữu và đặc điểm nông trại

1. Hệ thống nông trại (tích hợp/tách biệt)

☐ tích hợp ☐ tách biệt ☐ khác_______________________________________

2. Tổng diện tích nông trại

____________________ ha

3. Ông/bà quản lý/sở hữu nông trại này từ khi nào?

Tháng/Năm ______________________(Ví dụ 3/2002)

4. Tình trạng pháp lý đất

☐ Sổ xanh ☐ Sổ đỏ ☐ Giấy sở hữu nhưng chưa chính thức

☐ Quản lý cho bên thứ 3 ☐ khác____

5. Khi nào hết hạn hợp đồng ? *(ví dụ đến khi nào thì sổ xanh/đỏ được cấp)*

Tháng/năm ____________________(ví dụ 3/2002)

6. Khi ông/bà di chuyển đến đây, lý do chính là gì? *(có thể chọn nhiều đáp án)*

☐ nuôi tôm ☐ quản lý rừng ☐ gia đình/họ hàng ☐ thu nhập

☐ khác____

7. Ông/bà có thay đổi hệ thống nông trại kể từ khi ông/bà di chuyển đến đây (diện tích mặt nước, diện tích rừng)? *(có thể chọn nhiều đáp án)*

☐ không thay đổi ☐ trồng thêm rừng ☐chặt bớt rừng ☐ chuyển đổi giữa HT tích hợp/tách biệt
☐ tăng diện tích ao ☐ khác____

8. Lý do của những thay đổi trên là gì? *(có thể chọn nhiều đáp án)*

☐ diện tích ao tôm tăng lên ☐ quản lý dễ dàng hơn ☐ tối ưu hóa sản xuất

☐ thay đổi quy định ☐ tỷ lệ phủ rừng thì có lợi ☐ bảo tồn rừng ngập mặn
☐ khác____

Quản lý rừng ngập mặn

---

9. Tỷ lệ phủ rừng thực tế của nông trại là bao nhiêu?

________________% hoặc ________________ha trong tổng số ____________________ha

10. Quy định về tỷ lệ phủ rừng tối thiểu của nông trại là bao nhiêu?

☐ tôi không biết ☐ tôi biết, là ___________________% của tổng diện tích

11. Ông/bà có tuân thủ quy định này không?

☐ có ☐ không

12. Bao lâu thì cơ quan quản lý đi kiểm tra?

Một lần hoặc hơn mỗi: ☐ tháng ☐ 3 tháng ☐ 6 tháng ☐ năm

☐ ít hơn một lần/năm

13. Điều gì xảy ra nếu cơ quan quản lý phát hiện tỷ lệ che phủ rừng quá thấp? *(có thể chọn nhiều đáp án)*
☐ không có vấn đề gì ☐ phải đóng phạt ☐ phải trồng lại rừng ☐ chính quyền trồng lại rừng

☐ khác____

(-> nếu họ phải đóng tiền phạt, cố gắng tìm hiểu nó là bao nhiêu hoặc việc đền bù thế nào. Cần hết sức thận trọng. Số tiền: ____VNĐ)

14. ***Nếu ông/bà có quyền lựa chọn thì tỷ lệ che phủ rừng tốt nhất cho ông/bà là bao nhiêu?***

________________% hoặc ________________ha trong tổng số ____________________ha

15. ***Vì sao ông/bà nghĩ rằng đó là tỷ lệ tốt nhất?*** *(có thể chọn nhiều đáp án)*

☐ tổng thu nhập cao nhất ☐ sản lượng tôm cao nhất ☐ mang lại thu nhập tôi cần để sinh sống

☐ năng suất tối ưu ☐ dễ quản lý nhất ☐ cân bằng tốt giữa che mát với diện tích ao

☐ tốt nhất cho môi trường ☐ cân bằng tốt giữa các khía cạnh khác nhau (ví dụ thu nhập và môi trường)

16. ***Ông/bà sẽ thay đổi tỷ lệ che phủ rừng nếu quyền sở hữu thay đổi (ví dụ từ sổ xanh sang sổ đỏ)?***

☐ không ☐ có -> *vui lòng giải thích bằng cách đánh dấu vào một hoặc nhiều lựa chọn bên dưới*

☐ bảo tồn rừng ngập mặn lâu dài ☐ Tôi có thể kiếm thêm lợi nhuận từ gỗ

☐ quyền đàm phán mạnh hơn ☐ khác____

17. ***Ông/bà có cho rằng rừng ngập mặn tốt cho nông trại của mình không?***

☐ có ☐ không

Vui lòng giải thích tại sao____

18. ***Những lợi ích khác mà rừng ngập mặn mang lại là gì?***

☐ bảo vệ khỏi sóng thần ☐ đa dạng sinh học (chỗ trú cho động vật và thực vật) ☐ không có/vô ích

☐ điều hòa khí hậu ☐ nơi nuôi dưỡng tôm/cá ☐ gỗ và các mặt hàng khác

☐ vẻ đẹp/nhận dạng/nhà ☐ khác ____

19. ***Theo luật ông/bà cần phải duy trì ít nhất 50% rừng ngập mặn. Ông/bà nghĩ sao về mức giới hạn 50%?*** *(có thể chọn nhiều đáp án)*

☐ mức đó tốt ☐ chỉ có lợi cho môi trường ☐ tùy tiện/ngẫu nhiên ☐ mức đó <u>không</u> tốt

☐ không quan trọng, chỉ là một luật định ☐ nó khiến các cán bộ phân biệt đối xử đối với nông dân

Năng suất

20. ***Thu nhập bao nhiêu từ các sản phẩm thu hoạch?*** *(-> hỏi mỗi năm hay mỗi con nước; tính toán với họ)*

☐ Tôm sú _____________VNĐ ________________ / ___________________VNĐ mỗi năm

☐ Cua _____________VNĐ ________________ / ___________________VNĐ mỗi năm

☐ Cá _____________VNĐ ________________ / ___________________VNĐ mỗi năm

☐ Gỗ _____________VNĐ ________________ / ___________________VNĐ mỗi năm

☐ Khác _____________VNĐ ________________ / ___________________VNĐ mỗi năm

21. ***Ông/bà nhận xét năng suất hệ thống nuôi tôm của mình thế nào?***

☐tối ưu (không cần cải thiện) ☐ rất cao ☐ cao

☐ trung bình ☐ thấp ☐ rất thấp

22. Ông/bà có muốn cải thiện năng suất nuôi tôm của vuông mình không?

☐ có ☐ không

23. Ông/bà cải thiện năng suất tôm của vuông mình thế nào?

☐ tăng kích thước ao ☐ chặt thêm cây ☐ giống tốt hơn ☐ loài khác (ví dụ: cua)

☐ trồng thêm cây ☐ cải thiện quản lý (ví dụ các chu kỳ thả giống khác)

☐ khác____

Tham gia dự án tôm sinh thái Naturland hay các dự án tương tự và triển vọng trong tương lai

24. Ông/bà có tham gia dự án tôm sinh thái Naturland trước đây không (như của Seanamico)?

☐ có ☐ không *-> đi đến câu hỏi số 28*

25. Ông/bà đã tham gia vào dự án trong khoảng thời gian nào?

Bắt đầu ____(tháng/năm)

được chứng nhận ____(tháng/năm)

rời dự án ____(tháng/năm)

26. Mức độ hài lòng của ông/bà đối với dự án?

☐ rất nhiều ☐ trên trung bình ☐ trung bình ☐ không nhiều ☐ không hài lòng

27. a) Lý do ông/bà có nhận xét như trên? *(có thể chọn nhiều đáp án)*

☐ lợi nhuận khá ☐ chi phí lớn hơn lợi nhuận ☐ không có lợi nhuận tăng thêm

☐ dễ đáp ứng ☐ quản lý quá khó khăn ☐ quá nhiều thủ tục giấy tờ cho chứng nhận

☐ tôi có được kiến thức mới ☐ tôi muốn tham gia vào một dự án/nhóm

☐ khác____

b) Các hoạt động kiểm soát (khi có dự án) thì…… **…** *(-> chỉ chọn 1)*

☐ nhiều hơn ☐ tương tự ☐ ít hơn

28. Ông/bà có muốn tham gia vào dự án Naturland hay dự án tương tự khác không?

☐ có ☐ không ☐ có thể/không chắc ☐ tôi cần thêm thông tin để quyết định

29. Điều gì khiến ông/bà muốn tham gia vào dự án như vậy? *(có thể chọn nhiều đáp án)*

☐ giá tôm cao hơn, ngay cả nếu diện tích ao nhỏ hơn ☐ không thay đổi mật độ phủ rừng

☐ hỗ trơ kỹ thuật nuôi trồng ☐ tiếp cận con giống tốt hơn ☐ thành lập nhóm nông hộ

☐ tổng thu nhập của hộ cao hơn ☐ tham gia dự án cho phép tiếp cận nguồn vốn vay

☐ khác____

Triển vọng tương lai

30. Tầm quan trọng của các yếu tố sau đối với ông/bà?

Thu nhập ☐ rất quan trọng ☐ quan trọng ☐ hơi quan trọng ☐ không quan trọng

(Dễ) quản lý ☐ rất quan trọng ☐ quan trọng ☐ hơi quan trọng ☐ không quan trọng

(An toàn) tiếp cận thị trường ☐ rất quan trọng ☐ quan trọng ☐ hơi quan trọng ☐ không quan trọng

Tính linh hoạt (ví dụ: độ phủ rừng) ☐ rất quan trọng ☐ quan trọng ☐ hơi quan trọng ☐ không quan trọng

31. Ông/bà cho rằng mình sẽ ở đâu trong 20 năm tới? *(có thể chọn nhiều đáp án)*

☐ vẫn ở đây ☐ tôi không biết ☐ không còn ở đây nữa

☐ khác/giải thích____

32. Ông/bà có muốn con cái mình tiếp quản nông trại trong tương lai?

☐ có ☐ không ☐ tôi muốn chúng học và làm việc ở nơi khác ☐ tùy vào quyết định của chúng

33. Ông/bà có biết biến đổi khí hậu đề cập đến vấn đề gì không?

☐ có, tôi biết rất rõ ☐ không ☐ có thể/không chắc/hơi biết

34. Ông/bà đã từng được cơ quan quản lý hoặc chính quyền địa phương thông báo về biến đổi khí hậu và các tác động của nó chưa?

☐ có (vui lòng cho biết____) ☐ không

35. Ông/bà có tin rằng biến đổi khí hậu sẽ (hoặc đã) ảnh hưởng đến hoạt động nuôi trồng của mình trong tương lai không?

☐ có, nó sẽ có các tác động <u>xấu</u> ☐ có, nó sẽ có các tác động <u>tốt</u>

☐ không, nó sẽ không ảnh hưởng ☐ không chắc / có thể /không quan trọng

36. Ông/bà có tin rằng chính phủ Việt Nam và chính quyền địa phương áp dụng các biện pháp phù hợp nhằm giảm thiểu tác động xấu của biến đổi khí hậu đến mức tốt nhất có thể không?

☐ có ☐ không ☐ không chắc / có thể / tôi không biết

-> Xin lưu ý và thông báo cho người được phỏng vấn rằng thông tin này không cần thiết cho cuộc khảo sát. Nó có thể cung cấp thêm thông tin và giúp xác định các nông hộ trong trường hợp có nghi ngờ/chất vấn. Tuy nhiên, nếu người được phỏng vấn từ chối trả lời thì hãy dừng lại.

Ông/bà có câu hỏi nào không? *(-> hỏi họ xem có câu hỏi nào không hoặc có cần giải thích thêm không)*
Chúng tôi có thể hỏi thêm một số thông tin cá nhân cho việc thống kê và để làm rõ nếu chúng ta có bất kỳ câu hỏi nào khác sau này không? Đây hoàn toàn là tự nguyện và ông/bà không bắt buộc phải cung cấp thông tin này.

Cám ơn ông/bà đã tham gia !

Thông tin cá nhân

---

37. *Họ Tên*:_______________________________________

38. *Số tuổi*: ___________________________________

39. *Giới tính*: _________________________

40. ***Tổng số thành viên của hộ*** *(gồm chủ hộ)*:**__________________________**

41. ***Số điện thoại:***____

42. *Số thửa*: ____

Nội bộ

Tên người phỏng vấn_________________________________ Ngày: __________________________

Tỷ lệ che phủ rừng theo thông tin từ **BQLRPH**:________________% hoặc __________ha trong tổng số_____________ha

Ghi chú khác:
